# A consensus statement on a National Competency Framework for training and assessment of knowledge and skills in diabetes technologies, including hybrid closed loop (HCL), insulin pump systems, and continuous glucose monitoring (CGM) devices

**DOI:** 10.1111/dme.70253

**Published:** 2026-04-06

**Authors:** E. Richardson, B. Stribling, J. Ridgeway, C. Kitching‐Davis, L. Willcocks, L. Heggs, H. Wakefield, G. Gallen, A. Jolley, K. Bareford, A. Williams, D. Farrow, T. Fletcher‐Salt, D. Hicks, J. Higgins, L. Jordan, B. Kelly, C. Metcalf‐O’Shea, M. Turner, E. Walden, A. Walker, P. Choudhary

**Affiliations:** ^1^ University of Leicester Leicester UK; ^2^ University Hospitals Leicester Leicester UK; ^3^ Diabetes Technology Network London UK; ^4^ King's College Hospital London UK; ^5^ Diabetes UK London UK; ^6^ Diabetes Specialist Nurse Forum Harborough Enterprise Centre Market Harborough UK; ^7^ Breakthrough T1D London UK; ^8^ Trend Diabetes Harborough Enterprise Centre Market Harborough Leicestershire UK; ^9^ Royal College of Nursing London UK

**Keywords:** continuous glucose monitoring (CGM), diabetes, hybrid closed loop (HCL), technologies

## Abstract

**Aims:**

To design a national competency framework supporting the training of healthcare professionals (HCP) in the care and management of people with diabetes using diabetes technologies.

**Methods:**

Following the release of the NICE [TA943] guidance^1^ and the NHS England 5‐year implementation strategy^2^, a working group was formed to design a four‐level training framework for HCPs, identifying specific knowledge and competencies required for different roles in supporting people living with diabetes using continuous glucose monitoring (CGM) or hybrid closed‐loop systems.

**Results:**

We developed a four‐level training and competency framework using the ‘Novice to Expert’ framework and identified skills and competencies for each of those levels. We then sought endorsement from leading national organisations to create this consensus document. This framework complements the delivery recommendations of the 5‐year NHS implementation plan for HCL by helping HCPs develop competence and confidence to support HCL users.

**Conclusions:**

We anticipate that the national adoption of this training and competency framework will allow healthcare providers to assess and improve the care they provide. This in turn will improve access to therapies, care standards and improve clinical outcomes.

**What's new?**

- This consensus framework highlights different levels of knowledge and skills required by health care professionals (HCPs) in different roles to support people living with diabetes and using diabetes technologies.
- The framework enables HCPs and Services providers to identify appropriate levels of knowledge and skills required within different roles and indicates ways in which these may be ascertained.
- The framework will support HCPs and services to map the knowledge and skills they have in services in the use and management of diabetes technologies and identify ways in which they may positively influence the experience and care experienced by people living with diabetes.

## 1 BACKGROUND

Type 1 diabetes affects approximately 10% of the 537 million people living with diabetes worldwide and requires individuals to make continual adjustments to insulin delivery in response to food intake, physical activity and current glucose levels. Given the marked day‐to‐day variability in insulin requirements, it is unsurprising that many people with type 1 diabetes do not achieve the recommended glycaemic target.^1^ Continuous Glucose Monitoring (CGM) significantly improves diabetes care and management, with randomised controlled trials and real‐world studies demonstrating improvements in glycaemic control, reductions in acute diabetes complications^2^, ^3^ and enhanced quality of life.^4^, ^5^, ^6^, ^7^ The integration of some compatible CGM systems with insulin pump therapy has enabled the development of Hybrid Closed‐Loop (HCL) systems. The HCL system uses the CGM glucose values to automatically adjust insulin delivery given via an insulin pump and has shown incremental clinical benefits beyond those achieved with CGM and insulin pump therapy alone. Although, they still require the user to announce carbohydrate intake and activities.^8^

The wealth of evidence supporting the clinical and cost‐effectiveness of these diabetes technologies supported the development of the NICE TA943 and related best practice guidance.^9^, ^10^ This updated guidance recommends that hybrid closed‐loop systems be made available to all children and young people living with Type 1 Diabetes, all people planning pregnancy and all adults with Type 1 diabetes with an HbA1c of more than 7.5% (58 mmol/mol) who are already using CGM or pump therapy.

Historically, access to advanced diabetes technologies in the United Kingdom has varied substantially by locality and by the availability of local expertise.^11^, ^12^ However, recent national initiatives led by NHS England have sought to reduce this unwarranted variation by publishing a five‐year implementation strategy.^13^ This document anticipates the onboarding of more than 150,000 people to HCL therapy and supports the prioritisation of children, young people, pregnant women and those already using insulin pumps in line with the NICE TA943^9^ and has secured substantial dedicated funding to support its implementation.^13^ Despite the availability of resources that identify accessible technologies,^14^ it is recognised that there is an urgent and pressing need to provide robust, clinically relevant and cohesive training and assessment of clinical knowledge and skills.^11^, ^13^ The key overarching principle highlighted in this framework is the need for education and training to support the safe rollout of these technologies.

Although many healthcare professionals (HCPs) working in specialist diabetes services are now familiar with CGM, confidence and competence with HCL systems are less well established. Recent national surveys of UK clinicians have highlighted significant gaps in knowledge and confidence regarding HCL use, despite growth in both access and use.^15^, ^16^ This issue is particularly important as more people will require access and support using these systems.^13^ Clinicians must be able to assess suitability for these technologies, support shared decision‐making when selecting a system and optimise device settings to help individuals achieve their glycaemic targets. Clinicians must also be able to access and interpret data from dedicated platforms and proficiently manage common technical and clinical issues. Especially as the current HCL systems still require user interaction to inform the system of carbohydrate intake, activity, cannula and reservoir (or pod) replacement and recognise when the system is not functioning appropriately. Failure to identify problems such as infusion‐set occlusion or delayed responses to persistent hyperglycaemia (Glucose >16 mmol/L/288 mg/dL for ≥2 hours) may result in diabetic ketoacidosis^17^ and incorrect settings or delayed use of additional pump functions may increase the risks of ongoing or severe hypoglycaemia, underpinning the need for robust training and support.

The requirement for enhanced awareness and skills extends beyond specialist diabetes teams to other HCPs who care for people with type 1 diabetes. This includes colleagues in primary care, community services and HCPs working in non‐diabetes specialist areas in hospital settings.^18^ The National GIRFT reports and implementation strategies emphasise that service providers should ensure staff have adequate training and protected time to undertake this training to enable the maintenance of competencies that align with job roles and responsibilities, evolving technologies and standards of care.^11^

A review of available training provision across the United Kingdom identified a wide range of information resources (Appendix A). However, authors also noted that many were directed either at specific professional groups (such as doctors or nurses) or at people living with diabetes, limiting the access and relevance for people performing different clinical roles. Furthermore, despite national guidance promoting wider access to diabetes technologies and supporting implementation frameworks,^9^, ^13^ discussions in multiple national and international forums indicate that many HCPs working outside of the diabetes specialist teams remain unaware of the technologies (which are now considered standard of care^9^), the level of training they require or how to access the training required.

The proposed competency framework (Table 2) defines the essential knowledge and competencies required in four levels: awareness, competence, expertise and leadership (Table 1) and indicates the potential training required to meet them. It is hoped that this will also support training and educational providers in ensuring that resources, delivery methods and approaches to assessment align with each level of the framework. Adoption of this competency framework is also intended to help clinical teams identify and target training to the needs of specific staff groups. It also identifies opportunities for authentic assessment of knowledge and skills in line with national guidance,^19^, ^20^, ^21^, ^22^ fosters environments in which HCPs can develop and evidence their competencies in diabetes technologies in line with regulatory body requirements^23^, ^24^ and ultimately support people with diabetes to use CGM and HCL systems safely and effectively.

**TABLE 1:** Target audience for different levels of competence.

Level		Target audience
1	Awareness	Any HCP or support worker who, in the course of their duties, may come across people living with diabetes who may be using this technology
2	Competence	HCPs who support people with diabetes spend a significant portion of their time, and require competence in diabetes technologies to support them with basic management decisions and support with practical elements of care
3	Expertise	HCPs whose primary role includes supporting people using diabetes technology, who require a high level of expertise around the use of diabetes technologies and are competent to onboard patients, manage different clinical needs and support other HCPs develop their skills
4	Leadership	HCPs with expertise in diabetes technologies and who provide local or regional leadership in care delivery and complex case management

## 2 METHODS

This work was undertaken in a series of sequential phases.

### 2.1 Phase 1: Review of existing resources and correlated assessment modalities

Current educational resources and assessment modalities related to diabetes technologies were identified and reviewed to characterise current provision and approaches to competence assessment.

### 2.2 Phase 2: Definition of target audiences for each ‘level’

Target audiences for different levels of training were defined, and professional roles were mapped to these levels (Table 1) to ensure that the framework reflected the needs of diverse healthcare professionals.

### 2.3 Phase 3: Specification of knowledge skills and competency assessment

For each level, expected knowledge and skills were specified, allowing alignment of appropriate training activities to these expectations and the identification and development of authentic assessment methods to demonstrate attainment of the required competencies.

### 2.4 Phase 4: Collaboration and consensus

The draft framework was circulated to key stakeholders and subject‐matter experts in diabetes technology education, who were invited to provide feedback and indicate their willingness to support or affiliate with the framework as part of a consensus‐building process.

## 3 RESULTS

### 3.1 Phase 1: Review of existing resources and assessments

We performed a search of educational material, provided by different organisations such as the Diabetes Technology Network‐UK and EASD (Appendix A). We found that most of the training was developed with specific products in mind or was directed at specific audiences (i.e., specialists, HCPs working in primary/community care settings or people living with diabetes). Many of the resources did not specify the level of detail contained within the training modules or outline what competencies the trainee will develop by completing the training. This made it almost impossible to match this with the HCP's requirements according to job role and responsibilities. Whilst some of these resources did have end of module knowledge checks or assessments, these were not linked to different roles or requirements. Hence the desire to incorporate authentic assessment to support HCPs in demonstrating the application of learnt knowledge to their clinical practice through assessments of analysis and treatment decisions involving real‐life scenarios, particularly at higher levels of competency.

### 3.2 Phase 2: Identification of the target audience for each different level of competence (Table 1)

The range of HCPs whom a person living with type 1 diabetes might encounter, was mapped according to their varying roles, from those with minimal direct involvement to clinicians with primary responsibility for ongoing management and service organisation. These were then arranged into the four levels of competency: awareness, competence, expertise and leadership and expected knowledge, and each level was linked with a clearly defined set of expected competencies required to be able to deliver their role safely and effectively (Table 1).

#### 3.2.1 Level 1

This encompasses competencies for HCPs who are not routinely involved in day‐to‐day diabetes management but may see people with diabetes for other clinical reasons. These professionals require awareness of diabetes technologies, including recognition of potential indications for their initiation, some safety‐critical knowledge and the ability to signpost individuals to appropriate specialist services.

#### 3.2.2 Level 2

Identifies the competencies expected from clinicians who provide regular diabetes care, such as doctors, nurses and dietitians in primary or secondary care with a special interest in diabetes. These HCPs will require a basic level of knowledge and competence in diabetes technologies. This would include the ability to access and interpret relevant clinical data, and identify and manage common issues. This group would not be expected to be responsible for technology initiation or the management of highly complex cases.

#### 3.2.3 Level 3

Identifies the competencies required for HCPs who spend the majority of their time supporting people using diabetes technologies. These clinicians require detailed knowledge of available systems, their configuration and optimisation and their application in complex clinical scenarios.

#### 3.2.4 Level 4

Identifies the competencies required for those professionals who have attained Level 3 expertise and who also undertake leadership roles, including complex case management, service development, guideline implementation and strategic planning for diabetes technology services and provides direct input into the development of educational resources.

### 3.3 Phase 3: Development of the four‐level competency framework

A four‐level competency framework was developed, informed by national and professional guidance from Public Health England,^19^, ^20^ Trend Diabetes,^25^ the NMC^24^ and the GMC,^23^ to define the expected knowledge and skills required to attain each level and align these with appropriate training opportunities and authentic assessment strategies (Table 2). The authors were keen to integrate a variety of learning resources that actively incorporated Visual, Auditory, Reading/writing and Kinaesthetic (VARK) principles^26^ and fostered inclusive and authentic assessment,^27^, ^28^ into the framework to ensure it was able to accommodate different learning preferences, widen its appeal and ensure inclusivity for all HCPs.

**TABLE 2:** Knowledge, skills and competency assessment.

Level 1	Level 2	Level 3	Level 4
*Target audience* Any HCP whose primary role is not focussed on supporting people with diabetes, but who encounters people living with diabetes as part of their practice	*Target audience* HCPs who support people with diabetes as a significant portion of their time, and require competence in diabetes technologies to support them with basic management decisions and support with practical elements of care	*Target audience* HCPs actively engaged in diabetes technology on a day‐to‐day basis and who require expertise and knowledge of details of different systems and can provide in‐depth support, including on‐boarding and troubleshooting, to people using diabetes technologies	*Target audience* HCPs who have local or regional responsibilities for the delivery of diabetes technology care and are involved in service development and delivery as well as complex case management

Competencies			
**Understanding the systems**			
*Understand Insulin Pumps* What is an insulin pump? What does it do and not do? *Understand Continuous Glucose Monitoring (CGM)* What is a CGM? How do you get readings from it? *Understand Hybrid Closed‐Loop (HCL) Systems* What is a HCL system? What does it do and not do?	*Understand the different HCL and CGM systems* Be able to identify different systems and discuss basic differences between them Be able to signpost relevant supportive literature	*Understand and analyse the assessment process* Be able to assess a patient's suitability for diabetes technologies using supportive guidance and identify those who may not be suitable *Understand the current guidance* Identify the updated guidance and discuss how to apply it in difficult/different clinical scenarios *Understanding of the different systems* Discuss the differences between systems, how they work and their pros and cons	*Demonstrate service‐level Leadership* Provide the leadership skills required to effectively assess the skill mix needed to deliver diabetes services. To help improve quality and clinical outcomes leading to the publication of audit results, improvement in efficiency, innovation in clinical care and service development, management of obstacles and service evaluation *Demonstrate clinical‐level leadership* Discuss, practice and mentor staff in the management of complex situations, including exercise, pregnancy, End Stage Renal Failure (ESRF), Type 1 Disordered Eating (T1DE), gastroparesis and psychological distress related to diabetes and comorbidities
**Clinical application**			
*Awareness of eligibility for Hybrid Closed‐Loop Systems* Aware of eligibility for CGM and HCL systems *Understanding Blood Glucose (BG) and Ketone Levels* Understand BG and ketone levels	*Understand the management of the following situations/circumstances and describe the implementation actions required* Hypoglycaemia Hyperglycaemia Exercise Impact of carbohydrates Insulin pump/infusion set failure *Understand the information contained in downloaded data* Identify key information and calculate appropriate settings for diabetes technologies *Understand targets for HCL* Discuss when these may be altered *Understand local/national guidance* Describe actions required if an individual is admitted to acute sector for care (i.e., A&E or secondary care) *Understand specific guidance* on exercise and pregnancy	*Demonstrate System set up* Actively demonstrate the ability to program different systems and support in the on‐boarding of people living with diabetes onto HCL including appropriate choices of cannula (Steel/Teflon)/tubing/reservoir *Accurate Calculation of settings* Describe calculation of settings, the guidance used and calculate settings accurately *Identify and supply Supportive equipment* Identify appropriate devices for monitoring blood glucose and blood ketone levels in case of a sensor failure or concerns regarding erroneous readings *Sharing knowledge and skills* Support other members of the team to ensure they have appropriate competence *Accurately discuss nutritional impact* Identify situations where it is appropriate to provide tailored education that supports the nutritional requirements of individuals. Be able to discuss the glycaemic effects of different foods, alcohol, weight management and the concepts of carbohydrate counting or other supportive strategies where appropriate *Accurately interpret download data* Identify behavioural or structural (settings) issues within the download data and identify how they would assist the individual in the adjustment of settings within the HCL system to support the individual with the management of their diabetes	*Demonstrate advanced clinical application* Discuss the advanced clinical skills required to support individuals with diabetes in developing the self‐management skills required when using advanced technologies to manage their diabetes and demonstrate clinical application *Demonstrate advanced analytical skills* Demonstrate during practical sessions advanced analytical skills when interpreting downloaded data that supports complex care planning and discussions *Demonstrate advanced skills in providing education* Discuss how to tailor education to support informed decision making, regarding how this therapy differs when managing and monitoring their diabetes, including: specific insulin pump devices, follow‐up requirements, risk versus benefit and additional functions associated with HCL pump devices *Lead multi‐disciplinary teams* Bring together expertise within nurses, dietitians, psychologists and medical professionals to work together improve outcomes across the service
**Special situations**			
*Emergency Situations and Urgent Escalation* Identify emergency situations Take appropriate action or urgent escalation Awareness of situations where continuing HCL may not be appropriate and escalation is required Management of severe hypoglycaemia Recognition and initial management of Diabetic Ketoacidosis (DKA) *Emergency Protocols for Non‐communicative Individuals* In an emergency, check if the individual has additional devices stuck to their body Inform the senior nursing and medical team or contact the diabetes team	*Understand the impact of intercurrent illness*. Identify situations where HCL therapy is inappropriate *Identify possible structure or behavioural concerns* Understand how to set and correct common settings (insulin to carb ratios, insulin sensitivity factor, glucose targets, sensor alarms) Identify and troubleshoot common patterns that lead to low Time In Range (TIR) or high Time Below Range (TBR)	*Accurately identify Structure or behaviour*. Identify and describe common behaviours or structural issues that lead to low TIR and high TBR *Effectively manage an emergency intervention* Be able to describe how they would assist or provide advice and guidance to HCL users during illness, equipment failure or skin‐related issues, including action taken /onward referrals *Manage Exercise and Pregnancy* Be able to provide advice on the management of exercise and pregnancy	
**Signposting and supportive education**			
*Referral to Appropriate Services* Signpost individuals to appropriate services for advice and support	*Understand when to escalate to the specialist pump team*	*Identify Additional training* Identify additional resources to support themselves, mentor other HCPs and care workers to develop skills and knowledge in order to support individuals using insulin pump therapy, CGM and HCL therapy	*Demonstrate skills in Training /education and succession planning* Discuss how to deliver/develop supportive educational materials, direct individuals to supportive networks, peer support and practice models of diabetes care that foster empowerment and lifelong learning about diabetes

The framework specifies the core competencies required within each level and indicates possible training and educational activities to achieve them. It also aids in the identification of assessment methods or indicates ways this may be evidenced within routine clinical practice. Additionally, it is hoped that the integration of using a blend of face‐to‐face and virtual content delivery, including multiple‐choice questions, graphics and images, case‐based learning, checklists interactive exercises and reflection to assess competence and demonstrate transferability to clinical practice scenarios will support the development of transferable skills and ultimately improve care delivery.

### 3.4 Phase 4: Stakeholder review and consensus development

We approached other key stakeholders (including specialist nurses and medical professionals) and expert bodies (Breakthrough T1D, Diabetes Technology Network (DTN), Diabetes UK (DUK), DSN Forum, Royal College of Nursing (RCN) and Trend Diabetes) working in the field of diabetes to review the proposed competency framework. Comments from different stakeholders were reviewed and incorporated to produce this consensus statement. All stakeholders have fully endorsed the proposed framework and have provided formal affiliation agreements for this consensus document that we hope can now be used to help providers support staff in gaining, developing and maintaining the required knowledge and skills.

## 4 DISCUSSION

This paper summarises the approach to develop a national framework for training for HCPs to develop confidence and competence in the clinical delivery and use of CGM and hybrid closed‐loop devices. This framework was conceived in response to the gaps in training identified by the GIRFT report,^11^ and addresses the training needs of HCPs across the board to develop from ‘novice to expert’ in this area.^29^ We hope that the adoption of this framework could help improve care for people living with diabetes using technologies in a number of different ways.

From the perspective of the person living with diabetes, it is hoped that this framework may increase awareness of the level of knowledge relating to diabetes technologies and expectations of levels of knowledge that different HCPs may possess. From the healthcare professional perspective, it is hoped that this document will help HCPs identify training requirements related to their roles and responsibilities and include them in their personal development plans.

It is important to recognise that this framework doesn't necessarily suggest that all people will progress through the four levels of competency. For example, there are many very experienced clinicians who already possess skills and expertise relevant to level three and four, and there are many others who only need level one or level two competencies. If, however, there is someone who wants to progress, this framework makes it easy to identify the knowledge and competency they need to acquire and how they may be able to demonstrate their achievements.

Finally, from the perspective of the healthcare provider, it provides a framework in which an organisation could ensure that their services contain HCPs with the correct skill mix to provide effective, safe and efficient services. Additionally, it would support them in identifying where there is a gap between roles and competencies, thereby helping direct those staff to the right training opportunities.

The framework also has value for providers of training in diabetes technology as it would support trainers to target the education to the right level according to need. For example, the training required for a nurse in primary care might be at level one or level two, whilst training for specialists might be at level three or level four. The authors believe the main strength of this document is that it is the first document that explicitly identifies the level of competence required according to different roles and responsibilities within services, helping to identify training requirements and suggesting ways in which those competencies can be measured and attained. The endorsement and affiliation by all the leading professional bodies suggest that it presents face validity.

The authors expect that training for level 1 competencies could incorporate online modules, webinars or be done in the format of mandatory training, as is often done for insulin safety. Training for level 2 would be more detailed and may include some case studies and data interpretation. It is possible that this can be delivered online but may be better done face to face. We also suggest that Level 3 competencies will require direct handling and interface with the technology, as users need to be competent in ‘button‐pressing’ and device setup. Additionally, we expect that Level 4 competencies will be more unique, as they will depend on individual service requirements and current levels of expertise within teams.

The impact of the framework can only be measured following publication and implementation by healthcare providers, to scope out the gap between current knowledge and skills and the implementation benefits on service delivery. It is hoped that this framework will also impact the educational resources currently available through both industry and other providers to ensure that the training offered is targeted, clinically valuable and meaningful, and that knowledge and skills learned are appropriately assessed.

## 5 AREAS FOR FUTURE DEVELOPMENT

As with all guidance, it is acknowledged that the training resources and linked assessments will require regular updates, especially given the rapid evolution of clinical evidence and new innovations. We plan to review this annually (or earlier, if significant changes in guidance occur that would impact the competency framework content).

If this framework is adopted at a national level, it is hoped that data relating to knowledge and competence levels of the clinical teams could also be captured and provide a basis for analysis of any association with clinical outcome data. Ultimately the authors believe that it is the responsibility of healthcare providers to ensure that staff employed to perform specific roles have appropriate training and competencies to deliver those roles. We hope that this consensus, endorsed by all the major professional bodies, provides healthcare providers with the framework by which they can achieve this.

## 6 RECOMMENDATIONS

There is a clear indication of the need for national standardisation in training and assessment of knowledge and skills to reduce the risk of developing different care standards across the United Kingdom. Therefore, this framework will need to be adopted at a national level, implemented and evaluated to investigate the impact on HCPs' knowledge and skills, service delivery standards, and health outcomes. Our partner organisations, who have endorsed this framework, would need to champion clinical providers to ensure that HCPs in different roles have the right competencies, and providers of diabetes technology education would need to adapt their educational offerings to different levels to ensure that they are providing education appropriate to the right audience. This could then provide additional information to support national audits of diabetes services to ensure care delivered remains consistent regardless of location.

## 7 CONCLUSION

This paper describes the development of a 4‐level competency framework that specifies knowledge and competencies around diabetes technologies expected for HCPs in different roles who support people living with diabetes and using these devices. We anticipate that national adoption of this framework will improve access to diabetes devices, improve care standards, and ultimately improve clinical outcomes.

## FUNDING INFORMATION

This paper was produced without any direct financial support.

## CONFLICT OF INTEREST STATEMENT

The Leicester Diabetes Centre and the listed authors collaborate with various industry partners; however, no industry influence or input was involved in the creation of this position statement.

## APPENDIX A

**Table:**

Resource	Publication and date (s)	URL	intended audience	Linked to level
DTN‐UK Best Practice Guides	UK's Association of British Clinical Diabetologist's Diabetes Technology Network (ABCD‐DTN): Best practice guide for hybrid closed‐loop therapy (2023) Using diabetes technology in pregnancy (2020) Continuous subcutaneous insulin infusion (CSII) A clinical guide for adult diabetes services (2018) Guidelines for managing continuous subcutaneous insulin infusion (CSII, or ‘insulin pump’) therapy in hospitalised patients (2018) Continuous subcutaneous insulin infusion (CSII)—A guide to service requirements (2018)	https://abcd.care/current/dtn‐uk‐best‐practice‐guides	HCP	2,3,4
DTN‐UK educational videos	*Hybrid Closed Loops (HCL) Educational Videos* Expert Views on Devices Diabetes tech in pregnancy Flash Glucose Monitoring Continuous Glucose Monitoring (CGM) Virtual Consulting Self‐Monitoring Blood Glucose Connected Pens	https://abcd.care/dtn/education	HCP and people living with diabetes	1,2,3,4
Glooko Academy training videos and assessment	Glooko Academy (2022)	https://go.glooko.com/academy	HCP	2,3,4
Scoping Review and practical guidance document	Insulin Pumps and Hybrid Close Loop Systems Within Hospital: A Scoping Review and Practical Guidance From the Joint British Diabetes Societies for Inpatient Care (2022)	https://pmc.ncbi.nlm.nih.gov/articles/PMC10210119/	HCP	2,3,4
Leicester diabetes centre training	Practical Diabetes Technology & HCL Course (2025) Hybrid Closed‐Loop Technology Pathway Eden: 2 steps to glucose sensing (2020)	https://www.leicesterdiabetescentre.org.uk/practical‐diabetes‐technology‐course https://www.leicesterdiabetescentre.org.uk/4‐levels‐to‐diabetes‐technology https://www.edendiabetes.com/training2‐steps‐to‐glucose‐sensing	HCP HCP HCP	2,3 1,2,3,4 2
EASD training videos and assessment	Technology and type 1 diabetes (2025)	https://easd‐elearning.org/our‐courses	HCP	2,3
NHS England decision‐making tool	Making a decision about managing type 1 diabetes (2024)	https://www.england.nhs.uk/wp‐content/uploads/2024/01/PRN00250‐decision‐support‐tool‐making‐a‐decision‐about‐managing‐type‐1‐diabetes‐v2.pdf	HCP	1,2
CPD	Closed‐Loop Insulin Delivery Systems: Past, Present, and Future Directions (2022)	https://pmc.ncbi.nlm.nih.gov/articles/PMC9207329/	HCP and people living with diabetes	2,3,4
